# Assessment of the real-world safety profile of vedolizumab using the United States Food and Drug Administration adverse event reporting system

**DOI:** 10.1371/journal.pone.0225572

**Published:** 2019-12-04

**Authors:** Raymond K. Cross, Michael Chiorean, Francis Vekeman, Yongling Xiao, Eric Wu, Jingdong Chao, Anthony W. Wang

**Affiliations:** 1 University of Maryland School of Medicine, Baltimore, Maryland, United States of America; 2 Virginia Mason Medical Center, Seattle, Washington, United States of America; 3 Groupe d’analyse, Ltée, Montréal, Québec, Canada; 4 Analysis Group, Inc., Boston, Massachusetts, United States of America; 5 AbbVie Inc., North Chicago, Illinois, United States of America; Auburn University, UNITED STATES

## Abstract

Vedolizumab is the first gut-selective integrin blocker indicated for patients with Crohn's disease (CD) and ulcerative colitis (UC). This study aimed to examine the adverse events (AEs) profile of vedolizumab compared to anti-tumor necrosis factors (anti-TNFs) indicated for CD and UC using the FDA Adverse Event Reporting System (FAERS) database. AE reports with vedolizumab (5/20/2014–6/30/2015) and CD/UC-indicated anti-TNF drugs (adalimumab, infliximab, certolizumab pegol, and golimumab, during 8/1/1998–6/30/2015) as primary suspects were extracted from the FAERS database. AEs associated with vedolizumab were compared for signals of disproportionate reporting against anti-TNF drugs and all other drugs (1969–6/30/2015), using the proportional reporting ratio (PRR) and the empirical Bayesian geometric mean (EBGM) algorithms. The search retrieved 499 reports for vedolizumab and 119,620 reports for anti-TNFs, with 35.9% and 32.1% of these, respectively, being serious AEs. With the PRR approach, vedolizumab-associated reports had signals for 22 groups of AEs (9 were associated with serious outcomes) relative to anti-TNFs and had 34 signals relative to all other drugs. Signals detected included those reported as warnings in prescribing information and new AEs related to cardiovascular disease. Due to the voluntary nature of FAERS, this finding should be considered hypothesis generating (rather than hypothesis testing). Longer-term observational studies are required to evaluate the safety of vedolizumab.

## Introduction

Biologic drugs target specific components of the immune system and have revolutionized the treatment of inflammatory bowel disease (IBD).[1–3] Anti-tumor necrosis factor (TNF) agents, which include adalimumab (Humira^®^ [AbbVie, Inc., North Chicago, IL]), certolizumab pegol (Cimzia^®^ [UCB, Inc., Smryna, GA]), golimumab (Simponi^®^ [Janssen Biotech Inc., Horsham, PA]), and infliximab (Remicade^®^ [Janssen Biotech Inc., Horsham, PA]), have shown to be effective, have an acceptable safety profile, and have been the standard of care for nearly 20 years.[4–7] Recently biologic therapies with novel mechanisms of action such as vedolizumab (Entyvio^®^ [Takeda Inc, Tokyo, Japan]), the first gut-targeted integrin blocker, have entered the market and provide alternative treatment options for IBD patients.[3] While vedolizumab has demonstrated a favorable safety profile in randomized control trials and in early real-world studies, its safety profile has yet to be compared with anti-TNF therapies. While the development of new therapies to treat IBD is crucial to patients, it is important to place the safety of new therapies–especially those with novel mechanisms of action–in the context of existing therapies.

Anti-TNF drugs work by systemically suppressing the activity of the pro-inflammatory and pro-apoptotic TNFα cytokine,[8] and thus suppressing the activation of downstream immune responses.[8] By contrast, integrin blockers disrupt leukocyte migration to sites of inflammation, providing a more selective inhibition of the chronic inflammatory response in IBD.[9] Vedolizumab mainly impacts the immune response in the gut through its interaction with the gut-associated alpha4-beta7 integrin on the surface of memory T cells.[10, 11] Vedolizumab was approved in May 2014 for the treatment of moderate-to-severe IBD and is still the only gut-selective leukocyte migration inhibitor approved in the US for the treatment of both UC and CD.[10]

The balance between clinical benefit and possible risks is essential in determining optimal treatment choice.[12–14] While the safety profiles of anti-TNF drugs are well-established from both randomized clinical trials (RCTs)[15–17] and real-world studies,[18–21] to date, information on possible adverse events (AEs) after treatment with vedolizumab comes mainly from clinical trials such as the two Phase III trials for vedolizumab in patients with UC and CD (GEMINI 1 and 2),[22, 23] with limited information derived from a few real-world studies with small sample sizes or relatively short follow-up that assessed the effectiveness and safety of vedolizumab.[24–27]^,^[28] However, while RCTs are the gold standard for assessing the efficacy of drugs, they are not ideal for detecting rare safety events.[29] The main shortcoming of the RCT study design is its limited external validity namely due to its often short duration of follow-up, limited study population size, stringent entry criteria that often exclude patients with significant comorbidities, older age, real-world population heterogeneity, and an artificially high level of adherence to treatment.[30–33] As a result, infrequent serious adverse events (SAEs) are often discovered through voluntary reporting systems or from nonrandomized post-marketing studies.[32] Thus, real-world observational studies and mining of pharmacovigilance data are used to augment safety information derived from RCTs and assist in detecting possible areas of caution when using the drug of interest.[34, 35] The FDA Adverse Event Reporting Systems (FAERS) is a voluntary reporting system developed by the FDA for the purpose of post-marketing surveillance for all approved drugs and therapeutic biologics. It gathers reports of AEs voluntarily submitted by health care professionals and consumers (directly or through the manufacturer of the drug), which may contain AEs that were already observed in RCTs and others that may not have been detected during RCTs.[36] Statistical methods have been developed to allow the effective interpretation of findings based on voluntary reporting systems.[37–39]

The objectives of this study were to assess the rates of real-world AE reporting associated with vedolizumab using the FAERS database. Three specific objectives were addressed in this study. The first objective was to explore the real-world profile of reported AEs with vedolizumab, as the unique mechanism of action (MOA) of vedolizumab provides the opportunity to compare gut-selective with systemic immunosuppressive drugs. The second objective was to identify whether there are any reported AEs that are disproportionately associated with vedolizumab relative to anti-TNFs for the treatment of UC and CD. The third objective was to identify whether there are any reported AEs disproportionately associated with vedolizumab relative to all other drugs reported in the FAERS database.

## Materials and methods

### Data source

The FAERS is a spontaneous reporting database maintained by the FDA,[40] whose aim is to support the FDA’s post-marketing safety surveillance program for drug and therapeutic biologic products by collecting information on adverse drug reactions from two principal sources: (1) mandatory reports from pharmaceutical companies (who must report any AE within 14 days of becoming aware of the AE), and (2) voluntary AE reports from healthcare professionals, consumers, and manufacturers. The database has over 9 million reports, with the earliest entries dating from 1969. In recent years (since 2012), the FAERS database has captured over 1 million reports annually. The data structure of the database adheres to the safety reporting guidance issued by the International Conference on Harmonisation (ICH) E2B. The FAERS public database includes seven datasets containing demographic and administrative information of the AEs (date FDA received the report, AE date, patient age, gender, reporting country, etc.), pharmaceutical details (drug names, indications, role of drugs, etc.), AE information, source of reports, and patient outcome information. In the database, AE names and indications for drug use are coded using the Medical Dictionary for Regulatory Activities (MedDRA) preferred terms.[41] Drugs are recorded by valid trade names if available or by verbatim names. Drug roles are classified as primary suspect, secondary suspect, concomitant, or interacting. Serious outcomes attributed to AEs, including death, life-threatening event, hospitalization (initial and prolonged), disability or permanent damage, congenital anomaly/birth defect, required intervention to prevent permanent impairment/damage (devices), or other serious outcomes (important medical events), are also captured.[42] Other drug-related information (such as dosage, administration route, etc.) is recorded using verbatim text. The FAERS data used in this study were provided by DrugLogic, Inc. (Reston, VA), which cleaned, mapped, de-duplicated, and normalized the raw FAERS data. This data has been utilized in multiple post-marketing adverse event analyses.[43–45]

### Sample selection

Reports associated with vedolizumab or anti-TNF drugs (i.e., adalimumab, certolizumab pegol, golimumab, and infliximab) were extracted by searching the generic and branded drug names along with potential misspellings and abbreviations, which were generated in the database cleaning/normalization process. Specifically, reports for vedolizumab were extracted from the date of FDA approval (May 20, 2014) to June 30, 2015 (the most recent update of the FAERS database at the time the study was conducted). Reports for anti-TNFs (i.e., adalimumab, certolizumab pegol, golimumab, and infliximab) were extracted from August 24, 1998 (the date of approval of infliximab—the first anti-TNF drug for CD) to June 30, 2015. Only reports listing the agents of interest (i.e., vedolizumab or anti-TNFs) as the primary suspect were kept (88% of reports for vedolizumab and 97.6% of reports for anti-TNFs). All vedolizumab reports were included without respect to indication because vedolizumab is only indicated for IBD. As anti-TNFs are indicated for multiple non-IBD inflammatory conditions, only reports where these drugs were indicated for UC or CD were included to ensure a homogeneous comparison with vedolizumab. Reports for all other drugs that were recorded in the FAERS database were extracted from inception to date of study execution (i.e., from 1969 to June 30, 2015). Ustekinumab (approved for Crohn’s disease after the last date available in the dataset) and natalizumab (primarily a treatment for multiple sclerosis with a black box warning for progressive multifocal leukoencephalopathy) were not included in the analysis.

Reports that satisfied the above inclusion criteria and were associated with serious outcomes were extracted for the subgroup analyses.

All data accessible within the FAERS database are fully anonymized and comply with the patient confidentiality requirements of the Health Insurance Portability and Accountability Act (HIPAA). Ethics approval was not required as the research does not contain any new studies with human or animal subjects performed by any of the authors.

### Study outcomes

Grouped AEs associated with vedolizumab were analyzed. Specifically, all individual AEs based on MedDRA preferred terms[41] recorded on vedolizumab reports were first identified, and then the preferred terms belonging to the same MedDRA High Level Term (HLT) class were grouped to form 254 grouped AEs (S1 Appendix).

### Statistical analysis

AE characteristics and patient demographics were reported using descriptive statistics (i.e., frequency, proportion, mean, and standard deviation) for vedolizumab and anti-TNF reports, respectively. Data mining algorithms were then used to identify AEs associated with vedolizumab that were reported more frequently than expected using the information on all drugs of interest as a reference (i.e., the reports for vedolizumab and anti-TNF drugs for Objective 2, and the reports for vedolizumab and all other drugs in the FAERS database for Objective 3). Two FDA-recommended data-mining algorithms for the analysis of spontaneous reports were used in this study: the proportional reporting ratio (PRR) and the empirical Bayesian geometric mean (EBGM).[38, 39, 46]

The PRR is one of the most commonly used frequentist methods of measuring reporting disproportionality.[39] In this study, the PRR was calculated as the ratio of the reporting proportion of AEs associated with vedolizumab divided by the reporting proportion of AEs associated with the comparator (i.e., anti-TNF drugs or all the other drugs in the FAERS database). The reporting proportion of a given AE for vedolizumab and the comparator, respectively, was calculated by dividing the number of reports with the given AE by the total number of reports for the drug of interest. A signal of disproportionate reporting was defined as a PRR ≥2, the lower bound of the PRR confidence interval ≥1, an associated chi-square ≥4, and the number of events in each group (i.e., vedolizumab and the comparator) ≥3.[47]

The EBGM calculation is conceptually similar to that of the PRR, but incorporates Bayesian “shrinkage” to produce more robust and stable disproportionality results when there are limited data and small numbers of events.[38, 39] The EBGM was calculated using the Bayesian multi-item gamma Poisson shrinker method.[38] A signal of disproportionate reporting was defined as the EB05 (i.e., the lower bound of the 90% confidence limit of EBGM) for vedolizumab ≥2, the EB05 for vedolizumab greater than EB95 (i.e., the upper bound of the 90% confidence limit of EBGM) for the comparator, and the number of events in each group ≥3.

The same two disproportionality analysis measures (PRR and EBGM) were calculated to examine the presence of signals of disproportionate reporting for reports associated with serious outcomes only.

SAS version 9.3 (SAS Institute, Inc., Cary, NC) and R version 3.2.2 (R Foundation, Vienna, Austria)[48] were used to conduct the statistical analyses.

## Results

A total of 499 reports were identified in which vedolizumab was listed as the primary suspect drug. Patient demographics are presented in Table 1. Vedolizumab was used in 44.3% of reports for CD, 23.4% for UC, and in 28.2% of reports the indication was not specified. Overall, 35.9% of vedolizumab reports were associated with serious outcomes. Among the 499 reports, 459 different MedDRA preferred terms were recorded and a total of 254 grouped AEs were created and compared for disproportionate reporting between vedolizumab, anti-TNF drugs and all other drugs in the database.

**Table 1:** Characteristics of reports—all reports for vedolizumab and anti-TNF drugs from August 1998 to June 2015.

	All Reports			Reports with Serious Outcomes		
Characteristics	Vedolizumab (N = 499)	anti-TNFs (N = 119,620)	*P*-value	Vedolizumab (N = 179)	anti-TNFs (N = 38,346)	*P*-value
**Age**						
Patients with available information, N (%)	285 (57.1)	75,036 (62.7)	0.01	132 (73.7)	25,473 (66.4)	0.04
Mean (SD)	42.8 (16.9)	40.7 (17.4)	0.10	42.1 (17.6)	39.2 (18.3)	0.10
**Gender, N (%)**						
Patients with non-missing information	432 (86.6)	116,641 (97.5)	<0.01	162 (90.5)	37,755 (98.5)	<0.01
Female	260 (60.2)	72,483 (62.1)	0.40	86 (53.1)	21,545 (57.1)	0.31
Male	172 (39.8)	44,158 (37.9)		76 (46.9)	16,210 (42.9)	
**Reporting Country, N (%)**						
Patients with available information	302 (60.5)	113,435 (94.8)	<0.01	162 (90.5)	35,033 (91.4)	0.68
US	189 (62.6)	77,941 (68.7)	0.02	96 (59.3)	14,675 (41.9)	<0.01
Non-US	113 (37.4)	35,494 (31.3)		66 (40.7)	20,358 (58.1)	
**Drug Indication, N (%)**						
Crohn's disease	221 (44.3)	101,268 (84.7)	<0.01	94 (52.5)	33,013 (86.1)	<0.01
Ulcerative colitis	117 (23.4)	18,352 (15.3)		49 (27.4)	5333 (13.9)	
Inflammatory bowel disease	11 (2.2)			4 (2.2)		
Other^a^	151 (1.4)			32 (17.9)		
Product used for unknown indication	140 (28.2)			27 (15.2)		
Missing	3 (0.6)			1 (0.6)		
**Serious Outcome, N (%)**						
Yes	179 (35.9)	38,346 (32.1)	0.07	179 (100.0)	38,346 (100.0)	
No	320 (64.1)	81,274 (67.9)		-	-	

^a^ Other indications for the use of vedolizumab include: enterocolitis hemorrhage, gastroenteritis, graft versus host disease, and rectal hemorrhage.

TNF, tumor necrosis factors

A total of 119,620 reports were identified in which anti-TNF drugs were listed as primary suspects with indication for UC or CD (Table 1, left panel). Overall, 84.7% and 15.3% of all anti-TNF reports had an indication for CD and UC, respectively, and 32.1% were associated with serious outcomes. Vedolizumab users and anti-TNF users were similar in terms of age and gender (Table 1).

Table 2 presents the PRRs calculated for grouped AEs containing known AEs listed in the prescribing information for vedolizumab (i.e., nasopharyngitis, headache, arthralgia, nausea, pyrexia, upper respiratory tract infection, fatigue, cough, bronchitis, influenza, back pain, rash, pruritus, sinusitis, oropharyngeal pain, and pain in extremities). A total of 309 out of 499 of reported vedolizumab AEs occurred for the AEs listed in the prescribing information for vedolizumab. No PRR signals were detected among vedolizumab reports for any of these known AEs compared with anti-TNF drugs (Table 2, left panel). In comparison with all other drugs, 3 PRR signals were detected including joint related symptoms (e.g., arthralgia), upper respiratory tract infections (e.g., nasopharyngitis and sinusitis), and upper respiratory tract symptoms (e.g., oropharyngeal pain) (Table 2, right panel).

**Table 2:** Proportional reporting ratios for known adverse events for vedolizumab versus anti-TNF drugs or all other drugs^a^.

AEs Documented in PI for Vedolizumab	Grouped AE	Vedolizumab	anti-TNFs	PRR (95% CI)	Vedolizumab	Other drugs	PRR (95% CI)
		(N = 499)	(N = 119,620)		(N = 499)	(N = 9,522,121)	
Fatigue	Asthenic conditions	39 (7.8%)	9691 (8.1%)	1.0 (0.7, 1.3)	39 (7.8%)	608,345 (6.4%)	1.2 (0.9, 1.7)
Cough	Coughing and associated symptoms	5 (1.0%)	2629 (2.2%)	0.5 (0.2, 1.1)	5 (1.0%)	127,077 (1.3%)	0.8 (0.3, 1.8)
Pyrexia	Febrile disorders	22 (4.4%)	5030 (4.2%)	1.0 (0.7, 1.6)	22 (4.4%)	253,357 (2.7%)	1.7 (1.1, 2.5)
Headache	Headaches NEC	26 (5.2%)	4693 (3.9%)	1.3 (0.9, 1.9)	26 (5.2%)	321,318 (3.4%)	1.5 (1.1, 2.2)
Respiratory tract infection	Infections NEC	31 (6.2%)	12,313 (10.3%)	0.6 (0.4, 0.8)	31 (6.2%)	431,833 (4.5%)	1.4 (1.0, 1.9)
Arthralgia	Joint related signs and symptoms	37 (7.4%)	5298 (4.4%)	1.7 (1.2, 2.3)	37 (7.4%)	197,635 (2.1%)	3.6 (2.6, 4.9)*
Bronchitis	Lower respiratory tract and lung infections	13 (2.6%)	2935 (2.5%)	1.1 (0.6, 1.8)	13 (2.6%)	247,076 (2.6%)	1.0 (0.6, 1.7)
Back pain	Musculoskeletal and connective tissue pain and discomfort	22 (4.4%)	3,813 (3.2%)	1.4 (0.9, 2.1)	22 (4.4%)	278,808 (2.9%)	1.5 (1.0, 2.3)
Nausea	Nausea and vomiting symptoms	40 (8.0%)	7,514 (6.3%)	1.3 (0.9, 1.7)	40 (8.0%)	560,626 (5.9%)	1.4 (1.0, 1.8)
Pruritus	Pruritus NEC	16 (3.2%)	4,815 (4.0%)	0.8 (0.5, 1.3)	16 (3.2%)	265,036 (2.8%)	1.2 (0.7, 1.9)
Rash	Rashes, eruptions and exanthemas NEC	27 (5.4%)	5,856 (4.9%)	1.1 (0.8, 1.6)	27 (5.4%)	359,453 (3.8%)	1.4 (1.0, 2.1)
Nasopharyngitis Sinusitis	Upper respiratory tract infections	18 (3.6%)	4,371 (3.7%)	1.0 (0.6, 1.6)	18 (3.6%)	124,124 (1.3%)	2.8 (1.8, 4.4)*
Oropharyngeal pain	Upper respiratory tract signs and symptoms	13 (2.6%)	2,919 (2.4%)	1.1 (0.6, 1.8)	13 (2.6%)	98,271 (1.0%)	2.5 (1.5, 4.3)*

* indicates that there is a PRR signal.

AE, adverse event; CI, confidence interval; TNF, tumor necrosis factors; NEC, not elsewhere classified; PI, prescribing information; PRR, proportional reporting ratio.

^a^ The PRRs were calculated for the grouped AEs (i.e., the 2nd column) that contain the AEs (i.e., the 1st column) documented in PI for vedolizumab.

Among all 254 grouped AEs identified for vedolizumab in the FAERS database, PRR signals were detected for 22 when compared with anti-TNFs (Table 3), of which 7 also had an EBGM signal. These 22 grouped AEs include AEs related to cardiovascular disease (pulmonary thrombotic and embolic conditions, pulmonary edemas, as well as central nervous system hemorrhages and cerebrovascular accidents) and some AEs discussed in the Warnings and Precautions section of vedolizumab’s prescribing information (infusion site reactions, infections, liver abnormalities, and colorectal neoplasms) (Table 3, left panel). A total of 17 vedolizumab and 1074 anti-TNF-associated reports detailed an AE related to cardiovascular disease; most of these reports were associated with serious outcomes (88.2% and 85.7%, respectively). The safety signal for central nervous system hemorrhages and cerebrovascular accidents for vedolizumab compared with anti-TNFs were detected by both PRR and EBGM.

**Table 3:** Proportional reporting ratios of adverse events with a signal for vedolizumab compared with anti-TNF drugs.

AE	All Reports				Reports with Serious Outcomes			
	Number of AEs (percent)				Number of AEs (percent)			
	Vedolizumab (N = 499)	anti-TNFs (N = 119,620)	PRR (95% CI)	PRR Signal	Vedolizumab (N = 179)	anti-TNFs (N = 38,346)	PRR (95% CI)	PRR Signal
Colorectal neoplasms malignant	3 (0.6%)	24 (0.0%)	30.0 (9.1, 99.2)	Yes *	1 (0.6%)	11 (0.0%)	19.5 (2.5, 150.0)	No
Infusion site reactions	7 (1.4%)	58 (0.0%)	28.9 (13.3, 63.1)	Yes *	1 (0.6%)	16 (0.0%)	13.4 (1.8, 100.4)	No
Duodenal ulcers and perforation	3 (0.6%)	44 (0.0%)	16.3 (5.1, 52.5)	Yes *	3 (1.7%)	34 (0.1%)	18.9 (5.9, 61.0)	Yes *
Central nervous system and spinal infections	3 (0.6%)	46 (0.0%)	15.6 (4.9, 50.1)	Yes *	2 (1.1%)	38 (0.1%)	11.3 (2.7, 46.4)	No
Histoplasma infections	3 (0.6%)	84 (0.1%)	8.6 (2.7, 27.0)	Yes	3 (1.7%)	55 (0.1%)	11.7 (3.7, 37.0)	Yes *
Mental impairment (excluding dementia and memory loss)	5 (1.0%)	197 (0.2%)	6.1 (2.5, 14.7)	Yes *	0 (0.0%)	57 (0.1%)	0.0^a^	No
Liver function analyses	17 (3.4%)	795 (0.7%)	5.1 (3.2, 8.2)	Yes *	3 (1.7%)	373 (1.0%)	1.7 (0.6, 5.3)	No
Central nervous system hemorrhages and cerebrovascular accidents	9 (1.8%)	430 (0.4%)	5.0 (2.6, 9.7)	Yes *	8 (4.5%)	337 (0.9%)	5.1 (2.6, 10.1)	Yes
Pulmonary edemas	3 (0.6%)	160 (0.1%)	4.5 (1.4, 14.0)	Yes	3 (1.7%)	155 (0.4%)	4.1 (1.3, 12.9)	Yes
Urinary abnormalities	3 (0.6%)	164 (0.1%)	4.4 (1.4, 13.7)	Yes	2 (1.1%)	54 (0.1%)	7.9 (1.9, 32.3)	No
Bone related signs and symptoms	4 (0.8%)	251 (0.2%)	3.8 (1.4, 10.2)	Yes	1 (0.6%)	80 (0.2%)	2.7 (0.4, 19.1)	No
Ocular disorders NEC	4 (0.8%)	261 (0.2%)	3.7 (1.4, 9.8)	Yes	1 (0.6%)	56 (0.1%)	3.8 (0.5, 27.5)	No
Confusion and disorientation	6 (1.2%)	413 (0.3%)	3.5 (1.6, 7.8)	Yes	0 (0.0%)	209 (0.5%)	0.0^a^	No
Stomatitis and ulceration	7 (1.4%)	526 (0.4%)	3.2 (1.5, 6.7)	Yes	4 (2.2%)	213 (0.6%)	4.0 (1.5, 10.7)	Yes
Heart rate and pulse investigations	7 (1.4%)	539 (0.5%)	3.1 (1.5, 6.5)	Yes	4 (2.2%)	226 (0.6%)	3.8 (1.4, 10.1)	Yes
Sensory abnormalities NEC	5 (1.0%)	388 (0.3%)	3.1 (1.3, 7.4)	Yes	0 (0.0%)	89 (0.2%)	0.0^a^	No
Clostridia infections	7 (1.4%)	570 (0.5%)	2.9 (1.4, 6.2)	Yes	4 (2.2%)	367 (1.0%)	2.3 (0.9, 6.2)	No
Muscle pains	15 (3.0%)	1,401 (1.2%)	2.6 (1.6, 4.2)	Yes	5 (2.8%)	427 (1.1%)	2.5 (1.1, 6.0)	Yes
Viral infections NEC	5 (1.0%)	477 (0.4%)	2.5 (1.0, 6.0)	Yes	4 (2.2%)	244 (0.6%)	3.5 (1.3, 9.3)	Yes
Pulmonary thrombotic and embolic conditions	5 (1.0%)	484 (0.4%)	2.5 (1.0, 6.0)	Yes	4 (2.2%)	428 (1.1%)	2.0 (0.8, 5.3)	No
Dermal and epidermal conditions NEC	10 (2.0%)	1065 (0.9%)	2.3 (1.2, 4.2)	Yes	5 (2.8%)	242 (0.6%)	4.4 (1.8, 10.6)	Yes
Visual disorders NEC	9 (1.8%)	1000 (0.8%)	2.2 (1.1, 4.1)	Yes	1 (0.6%)	258 (0.7%)	0.8 (0.1, 5.9)	No

* indicates that the signal remains using the EBGM approach.

AE, adverse event; CI, confidence interval; TNF, tumor necrosis factors; NEC, not elsewhere classified; PRR, proportional reporting ratio.

^a^ The confidence interval was not estimated because there were no reported events for vedolizumab.

A total of 179 and 38,346 reports of AEs with serious outcomes were identified for vedolizumab and anti-TNFs, respectively (Table 1, right panel). Among the vedolizumab-associated reports with serious outcomes, the drug was used for CD in 52.5% and UC in 27.4% compared with 86.1% and 13.9% for anti-TNFs-associated reports, respectively. Of the 22 grouped AEs with a PRR signal identified using all eligible reports (Table 3, left panel), PRR signals were detected for 9 AEs using reports associated with serious outcomes, of which 2 also had an EBGM signal (Table 3, right panel).

In comparison with all other drugs reported in the FAERS database, the PRR analysis identified 34 signals for grouped AEs associated with vedolizumab. Of these, 14 also had an EBGM signal (Table 4, left panel). When restricting the analysis to reports with serious outcomes only, 21 of the 34 grouped AEs associated with vedolizumab had a PRR signal and 8 also had an EBGM signal (Table 4, right panel).

**Table 4:** Proportional reporting ratios for adverse events with a signal for vedolizumab compared with all other drugs.

AE	All Reports				Reports with Serious Outcomes			
	Number of AEs (percent)				Number of AEs (percent)			
	Vedolizumab (N = 499)	Other Drugs (N = 9,522,121)	PRR (95% CI)	PRR Signal	Vedolizumab (N = 179)	Other Drugs (N = 3,840,821)	PRR (95% CI)	PRR Signal
Histoplasma infections	3 (0.6%)	453 (0.0%)	126.4 (40.7, 392.0)	Yes *	3 (1.7%)	360 (0.0%)	178.8 (57.9, 551.8)	Yes *
Colorectal neoplasms malignant	3 (0.6%)	483 (0.0%)	118.5 (38.2, 367.5)	Yes *	1 (0.6%)	303 (0.0%)	70.8 (10.0, 501.6)	No
Central nervous system and spinal infections	3 (0.6%)	1604 (0.0%)	35.7 (11.5, 110.4)	Yes *	2 (1.1%)	1,413 (0.0%)	30.4 (7.6, 120.6)	No
Large intestine therapeutic procedures	5 (1.0%)	2936 (0.0%)	32.5 (13.6, 77.8)	Yes *	2 (1.1%)	2120 (0.1%)	20.2 (5.1, 80.4)	No
Infusion site reactions	7 (1.4%)	4316 (0.0%)	30.9 (14.8, 64.6)	Yes *	1 (0.6%)	1416 (0.0%)	15.2 (2.1, 107.1)	No
Gastrointestinal therapeutic procedures NEC	3 (0.6%)	2442 (0.0%)	23.4 (7.6, 72.5)	Yes *	1 (0.6%)	1695 (0.0%)	12.7 (1.8, 89.4)	No
Gastrointestinal fistulae	3 (0.6%)	2838 (0.0%)	20.2 (6.5, 62.4)	Yes *	1 (0.6%)	2003 (0.1%)	10.7 (1.5, 75.7)	No
Clostridia infections	7 (1.4%)	8293 (0.1%)	16.1 (7.7, 33.6)	Yes *	4 (2.2%)	6876 (0.2%)	12.5 (4.7, 32.9)	Yes *
Duodenal and small intestinal stenosis and obstruction	4 (0.8%)	8049 (0.1%)	9.5 (3.6, 25.2)	Yes *	4 (2.2%)	7313 (0.2%)	11.7 (4.5, 30.9)	Yes *
Soft tissue disorders NEC	9 (1.8%)	23,623 (0.2%)	7.3 (3.8, 13.9)	Yes *	3 (1.7%)	16,306 (0.4%)	3.9 (1.3, 12.1)	Yes
Colitis (excluding infective)	29 (5.8%)	82,006 (0.9%)	6.7 (4.7, 9.6)	Yes *	19 (10.6%)	54,632 (1.4%)	7.5 (4.9, 11.4)	Yes *
Skin structures and soft tissue infections	4 (0.8%)	12,255 (0.1%)	6.2 (2.3, 16.5)	Yes	3 (1.7%)	6177 (0.2%)	10.4 (3.4, 32.0)	Yes *
Intestinal ulcers and perforation NEC	5 (1.0%)	16,276 (0.2%)	5.9 (2.5, 14.0)	Yes	4 (2.2%)	14,144 (0.4%)	6.1 (2.3, 16.0)	Yes
Duodenal ulcers and perforation	3 (0.6%)	11,571 (0.1%)	4.9 (1.6, 15.3)	Yes	3 (1.7%)	10,126 (0.3%)	6.4 (2.1, 19.5)	Yes
Herpes viral infections	10 (2.0%)	38,860 (0.4%)	4.9 (2.7, 9.1)	Yes *	5 (2.8%)	17,337 (0.5%)	6.2 (2.6, 14.7)	Yes *
Gastrointestinal stenosis and obstruction NEC	11 (2.2%)	45,031 (0.5%)	4.7 (2.6, 8.4)	Yes *	8 (4.5%)	38,081 (1.0%)	4.5 (2.3, 8.9)	Yes
Viral infections NEC	5 (1.0%)	20,806 (0.2%)	4.6 (1.9, 11.0)	Yes	4 (2.2%)	13,706 (0.4%)	6.3 (2.4, 16.5)	Yes
Acnes	5 (1.0%)	20,837 (0.2%)	4.6 (1.9, 11.0)	Yes	2 (1.1%)	4747 (0.1%)	9.0 (2.3, 35.9)	No
Pulmonary edemas	3 (0.6%)	14,127 (0.1%)	4.1 (1.3, 12.5)	Yes	3 (1.7%)	13,451 (0.4%)	4.8 (1.6, 14.7)	Yes
Joint related signs and symptoms	37 (7.4%)	197,635 (2.1%)	3.6 (2.6, 4.9)	Yes *	11 (6.1%)	72,031 (1.9%)	3.3 (1.8, 5.8)	Yes
Psoriatic conditions	7 (1.4%)	39,729 (0.4%)	3.4 (1.6, 7.0)	Yes	2 (1.1%)	7769 (0.2%)	5.5 (1.4, 21.9)	No
Non-site specific procedural complications	4 (0.8%)	23,027 (0.2%)	3.3 (1.2, 8.8)	Yes	1 (0.6%)	10,661 (0.3%)	2.0 (0.3, 14.2)	No
Abdominal and gastrointestinal infections	10 (2.0%)	63,355 (0.7%)	3.0 (1.6, 5.6)	Yes	9 (5.0%)	48,595 (1.3%)	4.0 (2.1, 7.5)	Yes *
Dermal and epidermal conditions NEC	10 (2.0%)	64,425 (0.7%)	3.0 (1.6, 5.5)	Yes	5 (2.8%)	22,187 (0.6%)	4.8 (2.0, 11.5)	Yes
Ulcers NEC	19 (3.8%)	125,079 (1.3%)	2.9 (1.9, 4.5)	Yes	14 (7.8%)	80,330 (2.1%)	3.7 (2.3, 6.2)	Yes *
Stomatitis and ulceration	7 (1.4%)	47,477 (0.5%)	2.8 (1.3, 5.9)	Yes	4 (2.2%)	21,146 (0.6%)	4.1 (1.5, 10.7)	Yes
Upper respiratory tract infections	18 (3.6%)	124,124 (1.3%)	2.8 (1.8, 4.4)	Yes	5 (2.8%)	38,427 (1.0%)	2.8 (1.2, 6.6)	Yes
Therapeutic procedures NEC	7 (1.4%)	49,236 (0.5%)	2.7 (1.3, 5.7)	Yes	6 (3.4%)	36,385 (0.9%)	3.5 (1.6, 7.8)	Yes
Upper respiratory tract signs and symptoms	13 (2.6%)	98,271 (1.0%)	2.5 (1.5, 4.3)	Yes	4 (2.2%)	27,510 (0.7%)	3.1 (1.2, 8.2)	No
Bacterial infections NEC	7 (1.4%)	52,959 (0.6%)	2.5 (1.2, 5.3)	Yes	4 (2.2%)	39,759 (1.0%)	2.2 (0.8, 5.7)	No
Muscle pains	15 (3.0%)	116,337 (1.2%)	2.5 (1.5, 4.1)	Yes	5 (2.8%)	43,361 (1.1%)	2.5 (1.0, 5.9)	Yes
Gastrointestinal and abdominal pain^a^	31 (6.2%)	262,281 (2.8%)	2.3 (1.6, 3.2)	Yes	14 (7.8%)	120,746 (3.1%)	2.5 (1.5, 4.1)	Yes
Alopecia	10 (2.0%)	86,364 (0.9%)	2.2 (1.2, 4.1)	Yes	0 (0.0%)	13,315 (0.3%)	0.0^b^	No
Liver function tests	17 (3.4%)	147,909 (1.6%)	2.2 (1.4, 3.5)	Yes	3 (1.7%)	85,329 (2.2%)	0.8 (0.2, 2.3)	No

* indicates that the signal remains using the EBGM approach.

^a^ excluding oral and throat pain.

AE, adverse event; CI, confidence interval; TNF, tumor necrosis factors; NEC, not elsewhere classified; PRR, proportional reporting ratio.

^b^ The confidence interval was not estimated because there were no reported events for vedolizumab.

## Discussion

Safety is a crucial element of determining optimal treatment choice and is always a key concern when new therapies are introduced into the clinical practice. The safety profile of vedolizumab that has emerged from the analysis of clinical trial data in over 3,000 patients suggests an increased susceptibility to upper respiratory infections (nasopharyngitis, sinusitis), and other serious and opportunistic infections as well as non-specific AEs (nausea, fatigue, headache, arthralgia, rash, and pruritus).[9, 49–51] However, data from RCTs may underestimate the occurrence of rare but SAEs for which clinical trials have no adequate detection power due to sample size and relatively short-duration.[29] Post-marketing surveillance is currently required for all FDA-approved drugs so that rare AEs or AEs undetected in RCTs can be detected sooner after use in the real-world clinical setting.[36] Examples of examining potential novel side-effects associated with anti-TNF agents in post-marketing analyses of the FAERS database include the analysis of sporadic reports of non-Hodgkin's lymphoma[52–54] and of possible congenital anomalies (if the drug was administered to gestating patients).[55] The assessment of long-term safety of a drug is of the upmost importance to better educate patients and healthcare providers regarding the risks and benefits of treatment. Therefore, in the current study we have investigated the real-world safety profile of vedolizumab compared to anti-TNF and other drugs using the FAERS database.

In this study, no signals were detected for known AEs listed in vedolizumab’s prescribing information relative to anti-TNF use. However, signals were detected for arthralgia, nasopharyngitis, sinusitis, and oropharyngeal pain when comparing vedolizumab with all other drugs reported in the FAERS database.

Among the 254 grouped AEs identified for vedolizumab in the FAERS database, 22 and 34 were identified with signals of disproportionate reporting compared with anti-TNF drugs and all other drugs in the database, respectively. Grouped AEs with a PRR signal include AEs related to cardiovascular disease and AEs that had been reported in vedolizumab clinical trials and discussed in the Warnings and Precautions section of vedolizumab’s prescribing information (including infusion site reactions, infections, liver abnormalities, and colorectal neoplasms). AE signals detected in both comparisons include pulmonary edemas, infusion site reactions, infections, liver abnormalities, and colorectal neoplasms.

Several hypotheses exist for the signal detected for central nervous system hemorrhages and cerebrovascular accidents associated with vedolizumab relative to anti-TNF drugs. First, the reported cardiovascular disease AEs may be related to the different mechanisms of action of vedolizumab and anti-TNFs. Second, cardiovascular disease signals are commonly missed in IBD clinical trials as they are uncommon.[34] In the case of IBD, cardiovascular AEs may be linked to the underlying IBD: chronic low-grade inflammation has been associated with both venous and arterial thromboembolic events[56–60] and, overall, the development of cardiovascular disease.[56, 57, 61] An alternative explanation for the reported cardiovascular disease AEs could be that vedolizumab would be utilized in moderately to severely active CD and UC patients with who have had an inadequate response or intolerance to a TNF inhibitor or corticosteroids as indicated by the FDA. Such patients may be at greater risk for cardiovascular complications secondary to poor long-term control of disease. However, due to the nature of the voluntary AE reporting system and the types of data it collects, the study could not examine or control for various potential confounding factors which could influence the incidence of cardiovascular disease-related AEs. Such confounding factors include patient’s lifestyle habits (smoking, daily physical activity levels, diet), prior or concurrent treatments (e.g., NSAIDs), and other comorbidities (e.g., obesity, diabetes), among others.

Our study has several limitations. First, we relied on a database which is subject to incomplete and inconsistent reporting, as well as ascertainment bias. For example, missing information on indications for drug use or having multiple indications for a same drug might affect the identification of eligible reports included in the study. However, such ascertainment bias should be minimal as less than 2% of selected reports had either missing (for vedolizumab reports only) or multiple indications. Second, the Weber effect, or the phenomenon of increased reporting in the early years of a product’s launch, is often found in spontaneous reporting. Thus, we also included the early years after anti-TNF launches to account for this issue. It should be noted that we are analyzing reports from the first 13 months following vedolizumab approval compared with nearly 17 years of anti-TNF reports. However, the cardiovascular AEs we observed will not likely be impacted by the Weber effect since these AEs will likely be reported regardless of time from launch. Third, data quality greatly varies across reports and by the type of reporters (e.g., specialists, general practitioners, patients, pharmaceutical companies) and there is no internal or external validation. Fourth, since spontaneous reporting systems are numerator-based, they provide no denominator for the number of drug users. Due to this limitation, there is no way to factor in actual drug utilization and no way to calculate population based incidence rates from FAERS. Fifth, for some AEs with very small counts, the reporting rates estimated using the PRR approach might have large variations, which may result in false-positive signals (to limit false positives a minimum of three reported events were required to detect a signal in the current analysis). However, for all signals detected by the PRR approach, the EBGM approach, a method developed to produce more robust and stable disproportionality results when there are limited data and small numbers of events, was also used. It should be noted that there is no gold standard for data mining algorithms. An alternative approach such as reporting odds ratio (ROR) in contrast to PRR could be used to adjusted to different confounding factors in multivariate logistic regression [47]. However, as previously noted, there is limited information on other potential confounders, such as comorbidities, information on disease duration and severity, disease phenotype, surgical history, smoking history, and concurrent IBD treatment. Nonetheless, the FAERS database has successfully been used in previous analyses of real-world post-marketing pharmacovigilance studies.[53, 55, 62–65] Given these limitations, it is important to consider our results as hypothesis-generating and deserving of further study. Taken together, findings from this study detect signals of vedolizumab with an elevated reporting of certain AEs, including cardiovascular and thromboembolic disease, compared to anti-TNFs in IBD patients. These findings should be confirmed in further studies.

## Supporting information

S1 Appendix Supplemental Appendix A: Unique Reaction HLTs. (DOCX)
