# Supplementary material for: Assessment of the real-world safety profile of vedolizumab using the United States Food and Drug Administration adverse event reporting system
Source: PLoS One. 2019 Dec 4;14(12):e0225572. doi: 10.1371/journal.pone.0225572 (PMC6892509; doi:10.1371/journal.pone.0225572)
Supplement: S1 Appendix — (DOCX) [file pone.0225572.s001.docx]

Supplemental Appendix A: Unique Reaction HLTs

|  |  |
| --- | --- |
| 1 | abdominal and gastrointestinal infections |
| 2 | abdominal hernias nec |
| 3 | abortion related conditions and complications |
| 4 | abortions spontaneous |
| 5 | accelerated and malignant hypertension |
| 6 | accidental exposures to product |
| 7 | acnes |
| 8 | acute and chronic pancreatitis |
| 9 | administration site reactions nec |
| 10 | adverse effect absent |
| 11 | allergic conditions nec |
| 12 | alopecias |
| 13 | anaemias nec |
| 14 | anal and rectal pains |
| 15 | anal and rectal signs and symptoms |
| 16 | anaphylactic responses |
| 17 | angioedemas |
| 18 | anxiety symptoms |
| 19 | apocrine and eccrine gland disorders |
| 20 | appetite disorders |
| 21 | arterial inflammations |
| 22 | arterial therapeutic procedures (excl aortic) |
| 23 | arthropathies nec |
| 24 | asthenic conditions |
| 25 | bacterial infections nec |
| 26 | behaviour and socialisation disturbances |
| 27 | bile duct infections and inflammations |
| 28 | bladder and urethral symptoms |
| 29 | bladder neoplasms malignant |
| 30 | blindness (excl colour blindness) |
| 31 | bone and joint infections |
| 32 | bone related signs and symptoms |
| 33 | breast and nipple neoplasms malignant |
| 34 | breathing abnormalities |
| 35 | bronchospasm and obstruction |
| 36 | bullous conditions |
| 37 | bursal disorders |
| 38 | calcium metabolism disorders |
| 39 | candida infections |
| 40 | cardiac disorders congenital nec |
| 41 | cardiac infections |
| 42 | cardiac signs and symptoms nec |
| 43 | cartilage disorders |
| 44 | central nervous system and spinal infections |
| 45 | central nervous system haemorrhages and cerebrovascular accidents |
| 46 | cerebrovascular venous and sinus thrombosis |
| 47 | cholecystitis and cholelithiasis |
| 48 | cholestasis and jaundice |
| 49 | clostridia infections |
| 50 | colitis (excl infective) |
| 51 | colorectal neoplasms malignant |
| 52 | confusion and disorientation |
| 53 | connective tissue disorders |
| 54 | connective tissue disorders nec |
| 55 | coordination and balance disturbances |
| 56 | cortical dysfunction nec |
| 57 | coughing and associated symptoms |
| 58 | cytomegaloviral infections |
| 59 | death and sudden death |
| 60 | deliria |
| 61 | dental and oral soft tissue infections |
| 62 | depressive disorders |
| 63 | dermal and epidermal conditions nec |
| 64 | dermatitis ascribed to specific agent |
| 65 | diabetes mellitus (incl subtypes) |
| 66 | diarrhoea (excl infective) |
| 67 | disability issues |
| 68 | disorders of purine metabolism |
| 69 | disturbances in consciousness nec |
| 70 | disturbances in initiating and maintaining sleep |
| 71 | duodenal and small intestinal stenosis and obstruction |
| 72 | duodenal ulcers and perforation |
| 73 | dyspeptic signs and symptoms |
| 74 | dystonias |
| 75 | ear disorders nec |
| 76 | enterococcal infections |
| 77 | erythemas |
| 78 | exposures associated with pregnancy, delivery and lactation |
| 79 | eye and eyelid infections |
| 80 | faecal abnormalities nec |
| 81 | febrile disorders |
| 82 | feelings and sensations nec |
| 83 | female reproductive tract infections |
| 84 | flatulence, bloating and distension |
| 85 | foetal and neonatal conditions associated with product exposure |
| 86 | foetal and neonatal diagnostic procedures |
| 87 | foetal growth complications |
| 88 | fungal infections nec |
| 89 | gastric neoplasms malignant |
| 90 | gastrointestinal and abdominal pains (excl oral and throat) |
| 91 | gastrointestinal atonic and hypomotility disorders nec |
| 92 | gastrointestinal dyskinetic disorders |
| 93 | gastrointestinal fistulae |
| 94 | gastrointestinal signs and symptoms nec |
| 95 | gastrointestinal spastic and hypermotility disorders |
| 96 | gastrointestinal stenosis and obstruction nec |
| 97 | gastrointestinal therapeutic procedures nec |
| 98 | general nutritional disorders nec |
| 99 | general signs and symptoms nec |
| 100 | generalised tonic-clonic seizures |
| 101 | gestational age and weight conditions |
| 102 | haematological analyses nec |
| 103 | haematological disorders |
| 104 | haemorrhages nec |
| 105 | headaches nec |
| 106 | heart rate and pulse investigations |
| 107 | hepatic neoplasms malignant |
| 108 | hepatobiliary signs and symptoms |
| 109 | hepatocellular damage and hepatitis nec |
| 110 | herpes viral infections |
| 111 | histoplasma infections |
| 112 | hydrocephalic conditions |
| 113 | hyperacusia |
| 114 | hyperkeratoses |
| 115 | hypoglycaemic conditions nec |
| 116 | immune and associated conditions nec |
| 117 | infections nec |
| 118 | inflammations |
| 119 | infusion site reactions |
| 120 | injection site reactions |
| 121 | inner ear signs and symptoms |
| 122 | intervertebral disc disorders nec |
| 123 | intestinal haemorrhages |
| 124 | intestinal ulcers and perforation nec |
| 125 | iron deficiencies |
| 126 | ischaemic coronary artery disorders |
| 127 | joint related signs and symptoms |
| 128 | labour onset and length abnormalities |
| 129 | lacrimation disorders |
| 130 | large intestine therapeutic procedures |
| 131 | limb fractures and dislocations |
| 132 | liver function analyses |
| 133 | lower gastrointestinal neoplasms benign |
| 134 | lower respiratory tract and lung infections |
| 135 | lower respiratory tract inflammatory and immunologic conditions |
| 136 | lower respiratory tract signs and symptoms |
| 137 | lymphatic system disorders nec |
| 138 | magnesium metabolism disorders |
| 139 | maladministrations |
| 140 | medication errors nec |
| 141 | memory loss (excl dementia) |
| 142 | menstruation and uterine bleeding nec |
| 143 | mental disorders nec |
| 144 | mental impairment (excl dementia and memory loss) |
| 145 | migraine headaches |
| 146 | mineral and electrolyte analyses |
| 147 | multiple sclerosis acute and progressive |
| 148 | muscle pains |
| 149 | muscle related signs and symptoms nec |
| 150 | muscle weakness conditions |
| 151 | muscle, tendon and ligament injuries |
| 152 | musculoskeletal and connective tissue pain and discomfort |
| 153 | musculoskeletal and connective tissue signs and symptoms nec |
| 154 | nasal congestion and inflammations |
| 155 | nasal disorders nec |
| 156 | nausea and vomiting symptoms |
| 157 | neoplasms malignant site unspecified nec |
| 158 | neurological signs and symptoms nec |
| 159 | nocardia infections |
| 160 | non-site specific embolism and thrombosis |
| 161 | non-site specific gastrointestinal haemorrhages |
| 162 | non-site specific injuries nec |
| 163 | non-site specific procedural complications |
| 164 | noninfectious myocarditis |
| 165 | noninfectious pericarditis |
| 166 | normal pregnancy, labour and delivery |
| 167 | ocular disorders nec |
| 168 | oedema nec |
| 169 | off label uses |
| 170 | olfactory nerve disorders |
| 171 | oncologic complications and emergencies |
| 172 | optic disc abnormalities nec |
| 173 | oral soft tissue disorders nec |
| 174 | oral soft tissue swelling and oedema |
| 175 | pain and discomfort nec |
| 176 | panniculitides |
| 177 | paraesthesias and dysaesthesias |
| 178 | pelvic prolapse conditions |
| 179 | perception disturbances |
| 180 | peripheral embolism and thrombosis |
| 181 | peripheral neuropathies nec |
| 182 | peripheral vascular disorders nec |
| 183 | peripheral vasoconstriction, necrosis and vascular insufficiency |
| 184 | peritoneal and retroperitoneal disorders |
| 185 | pharyngeal disorders (excl infections and neoplasms) |
| 186 | physical examination procedures and organ system status |
| 187 | placental abnormalities (excl neoplasms) |
| 188 | pleural infections and inflammations |
| 189 | product use issues nec |
| 190 | prostatic neoplasms malignant |
| 191 | protein analyses nec |
| 192 | pruritus nec |
| 193 | pseudomonal infections |
| 194 | psoriatic conditions |
| 195 | psychotic disorder nec |
| 196 | pulmonary oedemas |
| 197 | pulmonary thrombotic and embolic conditions |
| 198 | rashes, eruptions and exanthems nec |
| 199 | rate and rhythm disorders nec |
| 200 | red blood cell analyses |
| 201 | renal disorders nec |
| 202 | renal lithiasis |
| 203 | reproductive tract signs and symptoms nec |
| 204 | respiratory syncytial viral infections |
| 205 | retinal bleeding and vascular disorders (excl retinopathy) |
| 206 | rotaviral infections |
| 207 | salivary gland infections and inflammations |
| 208 | salmonella infections |
| 209 | seizures and seizure disorders nec |
| 210 | sensory abnormalities nec |
| 211 | sepsis, bacteraemia, viraemia and fungaemia nec |
| 212 | site specific injuries nec |
| 213 | skin and subcutaneous tissue ulcerations |
| 214 | skin neoplasms malignant and unspecified (excl melanoma) |
| 215 | skin structures and soft tissue infections |
| 216 | sleep disorders nec |
| 217 | soft tissue disorders nec |
| 218 | soft tissue neoplasms benign nec |
| 219 | speech and language abnormalities |
| 220 | spinal fractures and dislocations |
| 221 | spleen disorders |
| 222 | staphylococcal infections |
| 223 | stomatitis and ulceration |
| 224 | suicidal and self-injurious behaviour |
| 225 | supraventricular arrhythmias |
| 226 | therapeutic and nontherapeutic responses |
| 227 | therapeutic procedures nec |
| 228 | thinking disturbances |
| 229 | thrombocytopenias |
| 230 | tic disorders |
| 231 | tissue enzyme analyses nec |
| 232 | tongue signs and symptoms |
| 233 | total fluid volume decreased |
| 234 | transient cerebrovascular events |
| 235 | tremor (excl congenital) |
| 236 | tuberculous infections |
| 237 | ulcers nec |
| 238 | upper respiratory tract infections |
| 239 | upper respiratory tract signs and symptoms |
| 240 | urinary abnormalities |
| 241 | urinary tract infections |
| 242 | urinary tract neoplasms malignant nec |
| 243 | urinary tract signs and symptoms nec |
| 244 | urticarias |
| 245 | vascular hypertensive disorders nec |
| 246 | vascular hypotensive disorders |
| 247 | vascular tests nec (incl blood pressure) |
| 248 | vascular therapeutic procedures nec |
| 249 | ventricular arrhythmias and cardiac arrest |
| 250 | viral infections nec |
| 251 | virus identification and serology |
| 252 | visual disorders nec |
| 253 | vulvovaginal disorders nec |
| 254 | white blood cell analyses |
